# Near-Field Vortex Beams Diffraction on Surface Micro-Defects and Diffractive Axicons for Polarization State Recognition

**DOI:** 10.3390/s21061973

**Published:** 2021-03-11

**Authors:** Dmitry Savelyev, Nikolay Kazanskiy

**Affiliations:** 1 Department of Technical Cybernetics, Samara National Research University, 443086 Samara, Russia; kazanskiy@ssau.ru; 2 Image Processing Systems Institute of RAS, Branch of the FSRC “Crystallography and Photonics” of the Russian Academy of Sciences (IPSI RAS), 443001 Samara, Russia

**Keywords:** optical vortices, diffractive axicons, surface micro-defects, FDTD, sensors

## Abstract

The diffraction of vortex Gaussian laser beams by elementary objects of micro-optics (surface micro-defects) to recognize the type of polarization (linear, circular, radial, azimuthal) of the input radiation was investigated in this paper. We considered two main types of defects (protrusion and depression in the form of a circle and a square) with different sizes (the radius and height were varied). Light propagation (3D) through the proposed micro-defects was modeled using the finite difference time domain (FDTD) method. The possibility of recognizing (including size change) of surface micro-defects (protrusions and depressions) and all the above types of polarization are shown. Thus, micro-defects act as sensors for the polarization state of the illuminating beam. The focusing properties of micro-defects are compared with diffractive axicons with different numerical apertures (NAs). The possibility of sub-wavelength focusing with element height change is demonstrated. In particular, it is numerically shown that a silicon cylinder (protrusion) forms a light spot with a minimum size of the all intensity FWHM of 0.28λ.

## 1. Introduction

The main property of singular light beams is the presence of a singular point at the wave front—a phase dislocation—which determines zero intensity [1]. A special place among such beams is occupied by beams with screw dislocations (optical vortices), which cause the vortex character of the propagation of light energy. Epy research of transformations of optical phase vortices and polarization singularities, and their mutual influence, has a long history [1,2,3,4,5,6,7,8]. The use of the vortex phase for solving the problems of analyzing the polarization properties of the laser field was proposed in [9,10,11,12]. The unusual properties of singular beams allow them to be used in a number of applications, including for optical manipulation of micro- and nanoparticles [5,6,7,8,13], material processing [14,15,16], and in microscopy [17,18,19]. Additionally, optical vortices are used to transmit information over fiber [20,21], in quantum informatics [22], and in wireless communication systems [23,24,25].

The introduction of a vortex phase singularity into the incident beam makes it possible to enhance the longitudinal component of uniformly polarized laser beams on the optical axis in the focal region [26], which makes it possible to change the diffraction pattern due to the redistribution of energy between the components of the electromagnetic field [27,28,29]. This possibility was experimentally confirmed in [30]. In the mentioned works, focusing elements with a refractive index *n* = 1.46 were considered. An increase in the refractive index allowed [26] to achieve an increase in the contribution of the longitudinal component to the overall intensity pattern on the optical axis. 

Beams of this kind can be generated using diffractive optics, such as spiral phase plates [31,32,33,34], spiral and twisted axicons [35,36,37,38], and multi-order diffractive optical elements [39,40,41,42,43]. The analysis of polarization features requires the use of devices based on interference schemes with anisotropic elements [44], and we can also use the correlation between polarization and phase features [45,46,47]. However, to recognize not only the differences between uniformly polarized and cylindrical beams, but also the direction of circular polarization and the difference between radial and azimuthal polarization, it is necessary to use sharp focusing [11]. In particular, the use of a high-aperture diffractive axicon for detecting such polarization states is known [12]. 

To obtain a sharp focus near the surface of optical elements, it is often sufficient to use such elementary micro-optics objects as microspheres, cylinders, individual steps [48,49,50], as well as arrays of these simple microelements (micro-cylinders, micro-holes) [51,52,53]. In particular, it was possible with use of them to achieve the size of the focal spot at the full width at half maximum (FWHM) of the intensity up to 0.38λ [51]. In [54], the focusing of light by a multilayer dielectric micro-cube (FWHM = 0.39λ) for the refractive index *n* = 2 was achieved. A study of the diffraction of a Gaussian beam on a separate cylinder with a sub-wavelength radius with a similar refractive index demonstrated that it is possible to achieve a decrease in the spot size according to FWHM to 0.36λ [55]. A further increase in the refractive index to 3.47 made it possible to focus the Gaussian beam near the element surface into a light spot, the size of which was FWHM = 0.25λ [56]. It was also demonstrated that a silicon cylinder illuminated by a laser beam with a first-order vortex phase singularity forms a light spot, the central part of which is mainly formed by the longitudinal component of the electric field (FWHM = 0.29λ) [56].

In this paper, we study the diffraction of vortex Gaussian laser beams by silicon surface micro-defects (*n* = 3.47) to recognize the type of polarization (linear, circular, radial, azimuthal) of the input radiation. We considered protrusions and depressions in the form of a circle and a square with different sizes (the radius and height were varied) as micro-defects. Additionally, the results of recognition of the above-mentioned polarization of laser radiation by diffractive axicons with numerical apertures (NAs) equal to 0.25 and 0.95 are presented for comparison. We wanted to determine the characteristics that provide subwavelength focusing, such as the type of relief features (protrusion or deepening), the size of the features (including variation not only in width but also in height), and the type of polarization of the vortex laser radiation and evaluate effect on the diffraction pattern of a separate protrusion close in size to the size of the central part of diffractive axicons with different numerical apertures. Numerical calculations of laser propagation (3D) were carried out using the finite difference time domain (FDTD) method using high-performance computer systems [57,58]. The calculations were performed on a computational cluster with a capacity of 850 Gflop.

## 2. Materials, Methods, and Simulation Parameters

Silicon (Si) is the second most abundant element (after oxygen) in the earth′s crust, and is a material with a high refractive index of *n* = 3.47. Currently, there are various fields of application of silicon and its compounds, in particular, for the manufacture of solar cells [59,60] and semiconductor devices (integrated circuits, diodes, transistors) [61,62], and for use in biology and medicine [63]. 

The height h of the relief of a binary element (even if it is a separate protrusion), corresponding to the phase jump π radians, for the selected refractive index is as follows:

(1) $$h=\frac{\pi }{k(n-1)}=0.202429\lambda \approx 0.2\lambda ,$$

where *k* = 2π/λ is wave number, λ is wavelength of laser radiation, and *n* is refractive index.

We considered both homogeneous polarization (linear and circular) of laser radiation and cylindrical polarization (radial and azimuthal).

It was shown earlier [26,29] that the addition of an optical vortex significantly changes the focal pattern, and the direction of rotation of circular polarization becomes important. At the second order of the optical vortex, and higher for “−”, circular polarization (the sign of circular polarization is opposite to the sign of the introduced vortex phase singularity), a shadow round light spot was formed. For this reason, in this paper, we consider the first order of an optical vortex in an incident beam. The polarization, the direction of which coincides with the direction of the vortex phase singularity, will be called “+”, namely circular polarization.

In this work, we consider the Laguerre–Gauss mode (0,1) as laser radiation with an introduced vortex (vortex phase change along the radius from 0 to 2π) phase singularity of the first order (m = 1):

(2) $$GL_{01}(r,\varphi ,z)=\left(\frac{\sqrt{2}r}{\sigma (z)}\right)\exp\left[ikz-i2\eta (z)\right]\exp\left[\frac{i\pi r^{2}}{\lambda R(z)}\right]\exp\left[-\frac{r^{2}}{\sigma^{2}(z)}\right]\exp\left(i\varphi \right).$$

where r^2^ = *x*^2^ + *y*^2^, φ = arctg(*y/x*), η(*z*) = arctg(*z*/*z*_0_), *R*(*z*) = z(1 + *z*_0_^2^/*z*^2^) is the radius of curvature of the parabolic front of the light field, σ(*z*) is the effective beam radius, *z*_0_ = πσ_0_^2^/λ is the confocal parameter, and λ is the wavelength of laser radiation.

It should be noted that the structure of the peripheral part of the zone plate with a short focus approaches the form of an axicon. In fact, the difference between the high NA axicon and the short focus zone plate is determined only by the central part. In particular, it was numerically shown in [58] that a two-zone lens illuminated by a laser beam with a first-order vortex phase singularity forms a light spot, the central part of which contains the longitudinal component of the electric field (minimum size FWHMz = 0.34λ). The total intensity of the light spot contains transversely polarized side lobes, which broaden the spot size to FWHM = 0.41λ. It was also numerically shown earlier that a separate cylinder with a subwavelength radius (refractive index *n* = 2) can be used to overcome the diffraction limit in the region of damped waves [55]. The minimum size of the light spot of a Gaussian beam was FWHM = 0.36λ. It was shown using the FDTD method that for a central zone radius of 0.1λ < r < 0.5λ, a focus is formed inside the optical element and only the energy of damped waves falls outside its boundary [55]. Thus, the influence of the size of the central part of the microelement is very important when focusing in the near field. 

The use of optical vortices and a diffractive axicon (*n* = 1.46) with a high numerical aperture (NA = 0.95) to analyze the polarization properties of the laser field was proposed in [12]. The plane wave expansion (PWE) method was used to describe focusing with an axicon. The ability to recognize the state of polarization under the condition of high numerical apertures of diffractive axicons has been demonstrated. In our case, the order of the optical vortex m is equal to 1, and the intensity on the optical axis at m = ±1 [12].

(3) $${\left|E(0,0,z)\right|}^{2}\approx \frac{{\left|c_{y}\mp ic_{x}\right|}^{2}}{4},$$

where (*c*_x_, *c*_y_) is the polarization vector for a homogeneous polarized beam. 

A zero value for a central focal spot indicates “+” is circular polarization, and a non-zero value indicates “−” is circular polarization [12]. 

Additionally, for cylindrical types of polarization at high values of the numerical aperture, at m = ±1, we obtain [12]:

(4) $${\left|E(0,0,z)\right|}^{2}\approx {\left|c_{\varphi }\right|}^{2},$$

where (*c*_r_, *c*_φ_) is the polarization vector in cylindrical coordinates. 

A zero value at the center focal point indicates radial polarization, and a nonzero value indicates azimuthal polarization [12].

It should be noted that it is quite possible to expect that the result of the calculation according to the rigorous electromagnetic theory will differ markedly from the results obtained on the basis of geometric optics or in the model of a thin element according to the Kirchhoff formulas. More precisely, the following can be said: In the framework of the geometrical–optical approach, a binary element at normal incidence of the beam does not change the incident beam at all, regardless of the relief height. When using the Kirchhoff integral (both in scalar and vector versions), only the phase incursion created by an element whose longitudinal size is considered to be small enough is taken into account.

**Figure 1:**
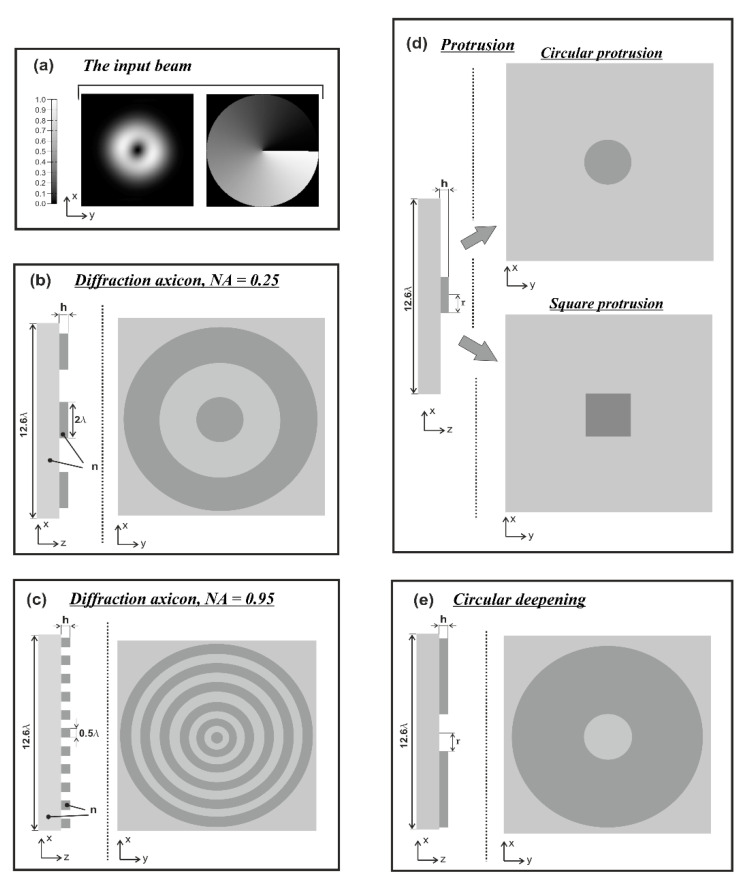
Input laser radiation and considered optical elements: input beam (**a**), diffractive axicon with NA = 0.25 (**b**) and NA = 0.95 (**c**), circular and square protrusion (**d**), circular deepening (**e**).

In this work, we considered silicon micro-cylinders (in the form of a protrusion and a depression), as well as a protrusion with a cross-section in the form of a square for analyzing the polarization properties of the laser field. Additionally, we presented the result of an analysis of the polarization properties of diffractive axicons with a numerical aperture NA = 0.25 (with a radius r of the central zone similar to the radius of a micro-cylinder, r = λ) and NA = 0.95 for comparison. As shown above (Equations (3) and (4)), for the high-aperture axicon, it was assumed that the considered types of polarization of the input laser radiation were accurately recognized. The considered elements and the input beam are shown in Figure 1.

Modeling parameters: radiation wavelength λ = 1.55 µm, size of the computational domain x, y, z in the range [−5.8λ; 5.8λ]. The thickness of the absorbing layer (PML) on all sides surrounding the computational domain is 1.16λ, the spatial sampling step is λ/30, and the time sampling step is λ/(60c), where c is the speed of light. The source is located inside the substrate, which occupies the entire space below the relief (Figure 1) and is partially embedded in the PML layer. The refractive index of the element is *n* = 3.47.

## 3. Investigation of the Laguerre–Gauss Mode (1,0) Diffraction by Surface Micro-Defects and Diffractive Axicons 

In this section, the conducted research of vortex Gaussian laser beam focusing by surface micro-defects and diffractive axicons is discussed explicitly.

### 3.1. Focusing by Micro-Defects 

Lithography is one of the main methods for obtaining integrated circuits and binary reliefs. It is based on the formation of a micro-relief in the photoresist, which provides the possibility of subsequent etching of the substrate material. As a result, a negative or positive micro-relief is formed [64]. It should be noted that in order to reduce the transverse dimensions of the micro-relief details in [64], it is proposed to use destructive interference due to a phase shift by π radians. The presence of such a phase dislocation in the wavefront leads to the formation of zero intensity in this region of space [65,66].

The silicon micro-cylinders discussed in the previous section can be made in two versions—in the form of a depression or a protrusion. In this case, as shown by the studies of this section, a completely different diffraction pattern was formed.

It was previously shown that it is possible to recognize both homogeneous and cylindrical types of polarization using diffractive axicons with NA = 0.95 (refractive index n = 1.46) [12]. The results of a study on the passage of the Laguerre–Gauss mode (1,0) through the surface micro-defects considered in the previous section (Figure 1d,e) are shown in the Table 1. The relief height was h = 0.2λ (Equation (1)) for x-linear, y-linear, “−” circular, “+” circular, radial, and azimuthal polarization of laser radiation (total intensity |**E**|^2^ is shown). We considered silicon cylinders (protrusion) with a radius of r = λ and r = 2λ, a depression with a radius of r = 2λ, and a square protrusion with r = 2λ (the side width of the square is 4λ).

In all considered cases, as can be seen from Table 1, we observed a strong difference in the formation of the diffraction pattern by different types of relief. For all types of polarization, except for circular polarization, intensity oscillations were observed. 

The focusing was observed in the near diffraction zone on silicon cylinders, and an increase in the element radius led to better focusing. A visual difference was observed between homogeneous and cylindrical polarizations; at a distance greater than λ for uniform polarization, a ring with zero intensity was formed on the optical axis, and for cylindrical types, the formation of a long light needle was observed. A similar result was observed for a square protrusion and a circular depression. Also noteworthy was the formation of an interference pattern from the edges of the depression. 

Nevertheless, at a distance smaller than λ, the visual picture is different for all considered types of polarization. The distance from the edge of the microrelief to the section plane is denoted as *z*_1_. Let us consider transverse diffraction patterns (*xy* plane) for all types of microdefects under study at a distance of *z*_1_ = 0.5λ. The results are shown in Table 2. Additionally, we assessed focusing by the considered types of elementary micro-elements. The focal spot size on the optical axis was estimated from the FWHM.

As can be seen from Table 2, we could visually distinguish all the considered types of polarizations. It was also possible to unambiguously determine the type of surface micro-defect: square or round protrusion and depression. 

Linear polarization for cylindrical protrusion and deepening was characterized by an elongated light spot along the corresponding polarization. 

Sections in the xy plane for x-linear and y-linear polarizations differed in that they were rotated 90 degrees (perpendicular) for the same micro-defects. The narrowest focal spot along one axis was obtained for x-linear polarization at r = 2λ (FWHM = 0.34λ).

A zero value for a central focal spot indicates “+” circular polarization, and a nonzero value indicates “−” circular polarization (Equation (3)). This fact is confirmed for all considered types of micro-defects. The most compact focal spot was obtained for “−” circular polarization at r = 2λ (FWHM = 0.45λ).

Equation (4) implies that a zero value at the center focal point also indicates radial polarization (row 5 of Table 2), and a nonzero value means azimuthal polarization (row 6 of Table 2). The most compact focal spot was obtained for azimuthal polarization at r = 2λ (FWHM = 0.47λ).

It is also worth noting that for silicon cylinders with “−” circular polarization, an intensity peak (near 1, i.e., near the maximum value) was formed on the focal axis, while for other polarizations, where focusing occurred, the intensity value of the central light spot was lower; for radial polarization at r = 1, an intensity of 0.32 was observed, while at r = 2, an intensity of 0.45 was observed for linear polarization—about 0.6 of the maximum intensity.

The central peak was formed for a square protrusion with “−” circular polarization, but its value was less than 0.2 of the maximum intensity. Nevertheless, if we consider the value at *z*_1_ = λ, then a central peak was formed with an intensity near 0.2 and FWHM = 0.46λ.

A peak formed on the optical axis for a circular deepening with azimuthal polarization, but its intensity value was only 0.12 of the maximum. The intensity value of the central focal spot for this type of micro-defect (“−” circular polarization) was 0.53.

**Figure 2:**
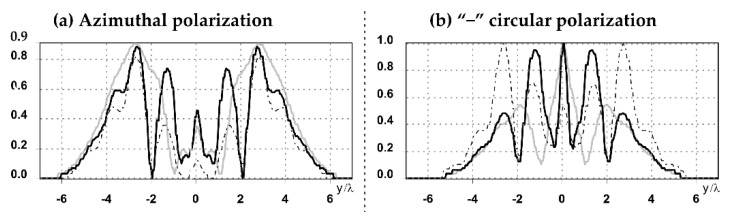
The cross sections for the cases in Table 2, circular protrusion with r = 2.0λ (black line), circular protrusion with r = 1.0λ (gray line), circular deepening (dash–dotted line): (**a**) azimuthal polarization, (**b**) “−” circular polarization.

The graphs with cross-sections for a circular protrusion and deepening for azimuthal and “−”—circular polarizations at a distance from the edge of the relief *z*_1_ = 0.5λ are shown in Figure 2. 

It is clearly seen in Figure 2 that the side lobes for azimuthal polarization were observed to be higher in intensity than for “−” circular polarization. The better focusing properties of the circular protrusion compared to the circular deepening were also clearly visible.

Thus, the result obtained in [12] for high-aperture diffractive axicons was demonstrated for elementary objects of micro-optics, in particular microcylinders. In the next section, we analyze polarization recognition using silicon diffractive axicons. 

### 3.2. Focusing by a Diffractive Axicons with Different Numerical Aperture 

One of the effective applications of diffractive microelements is the focusing of laser radiation in the near diffraction zone. The phase diffraction axicon has a complex transmission function of the following form:

(5) $$\tau (r)=\exp(ik\alpha_{0}r),$$

where *k* is wave number, λ is wavelength of laser radiation, and α_0_ is a parameter equal to the sine of the angle of the outgoing rays, depending on the material of the axicon and the angle at its apex. 

Equation (5) remains valid for a diffractive axicon with a continuous (disregarding jumps of 2π) phase change, but here α_0_ can be greater than unity. Below we consider the action of the simplest implementation in the form of a binary diffraction axicon, in which the phase takes on values of 0 and π radians.

We considered diffractive axicons (Figure 1b,c) with numerical apertures equal to 0.25 (grating period 4λ) and 0.95 (grating period 1.05λ). The radius of the central zone of the axicon with NA = 0.25 is equal to the radius of the central zone of the previously considered cylinder with r = λ. The results of numerical simulations for a diffractive axicon at low (NA = 0.25) and high (NA = 0.95) numerical apertures are shown in Table 3. We also considered the transverse diffraction patterns (xy plane) for the studied micro-axicons at a distance of *z*_1_ = 0.5λ (similar to Table 2). 

As can be seen from Table 3, we could visually distinguish all the considered types of polarizations, even for an axicon with a low numerical aperture, although in this case, for the azimuthal polarization, the central intensity peak was small. Equations (3) and (4) were also confirmed. It should be noted that for an axicon with NA = 0.25 farther λ from the element, only homogeneous and cylindrical polarizations could be recognized.

The diffractive axicon with NA = 0.95 gave better focusing and recognition, as expected. For linear polarization, the narrowest focal spot along one axis was obtained for x-linear polarization: FWHM = 0.43λ. The most compact focal spot was obtained for “−” circular polarization (FWHM = 0.48λ) and radial polarization (FWHM = 0.47λ).

It should be noted that earlier, for linear polarization for a silicon cylinder with r = 2λ, a narrower focal spot along one axis was obtained (FWHM = 0.34λ), for “−” circular polarization, the size of the focal spot was also slightly smaller (FWHM = 0.45λ), and for azimuthal polarization, the size was comparable.

The results were comparable for a silicon cylinder with r = λ and a diffractive axicon with NA = 0.25 for uniform types of polarization. Better discrimination between radial and azimuthal polarizations, as well as better focusing, was demonstrated by a silicon cylinder. In the case of a diffractive axicon, the intensity value on the focal axis (azimuthal polarization) was only 0.2 of the maximum intensity.

**Figure 3:**
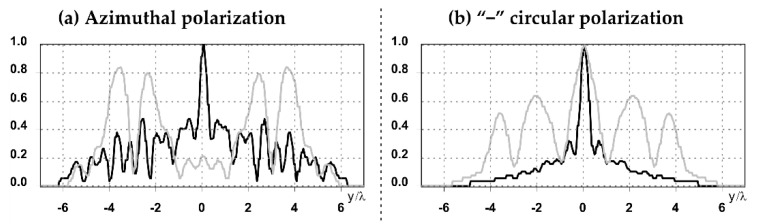
The cross sections on distance *z*_1_ = 0.5λ, diffractive axicon with NA = 0.95 (black line) and diffractive axicon with NA = 0.25 (gray line): (**a**) azimuthal polarization, (**b**) “−” circular polarization.

The graphs with cross-sections for diffractive axicon with NA = 0.25 and NA = 0.95 for azimuthal and “−” circular polarizations are shown in Figure 3. 

It is clearly seen in Figure 3 that a more pronounced focal spot with a higher intensity and smaller side lobes was observed when focusing with a diffractive axicon with NA = 0.95 than for focusing with a diffractive axicon with NA = 0.25.

Nevertheless, the intensity values of the central focal spot for circular polarization for both considered axicons were comparable. However, the focal spot was narrower for a diffractive axicon with NA = 0.95 for both considered cases.

**Figure 4:**
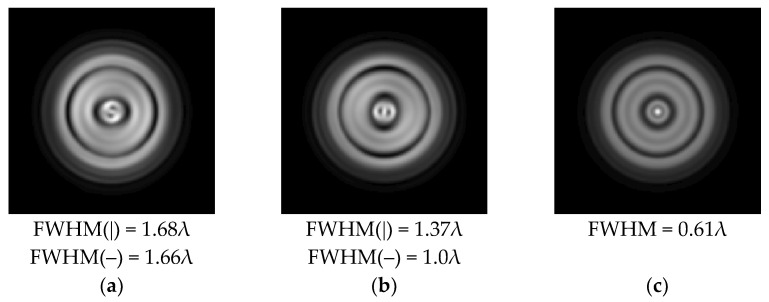
The diffraction of Laguerre–Gauss mode (0,1) with homogeneous polarization by a diffractive axicon with NA = 0.25, *z*_1_ = 0.2λ, polarization: (**a**) x-linear, (**b**) y-linear, (**c**) “−” circular.

It is also worth noting that for a diffractive axicon with NA = 0.25 for a homogeneous polarization near the element (*z*_1_ = 0.2λ), better focusing was observed than at a distance of *z*_1_ = 0.5λ (Figure 4).

**Figure 5:**
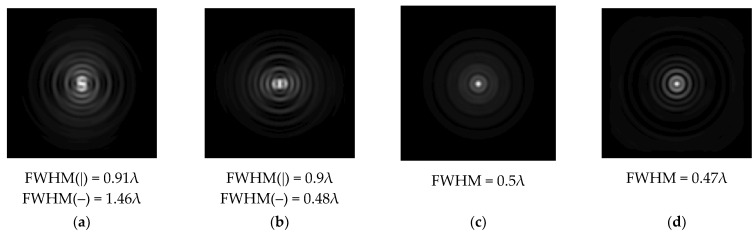
The diffraction of Laguerre–Gauss mode (0,1) by a diffractive axicon with NA = 0.95 in plane of maximum intensity, polarization: (**a**) x-linear, (**b**) y-linear, (**c**) “−” circular, (**d**) radial.

For a diffractive axicon with NA = 0.95, recognition was possible up to *z*_1_ = 3λ. Consider the cross-sections in terms of maximum intensity (more than 0.5λ) on the optical axis (Figure 5). It should be noted that for radial polarization, the size of the central focal spot did not change; for “−” circular, the size of the focal spot was comparable, and for linear polarization, a wider focal spot was obtained.

Thus, in this section, we showed the use of diffraction axicons to recognize the type of polarization (including the axicon with NA = 0.25). A silicon cylinder made it possible to obtain better or comparable focusing at a distance of *z*_1_ = 0.5λ than a diffractive axicon, including with NA = 0.95. We further varied the height of the considered optical elements. 

### 3.3. The Subwavelength Focusing with the Height Change of the Optical Elements 

Previously, the effect of changing the substrate on the diffraction pattern of a limited plane wave by diffractive axicons with different numerical apertures was shown, where the substrate thickness varied from 0.2λ to 0.3λ [26]. We fixed the type of polarization (“−” circular) and varied the height of micro-defects (from 0.2λ to 2λ). The research results (xz plane) are shown in Table 4. 

We considered the “−” circular polarization of the Laguerre–Gauss mode (0,1) due to the formation of a symmetrical spot with high intensity (Table 1). After choosing the optimal height from the point of focusing properties, we considered the remaining types of polarizations. The FWHM values were taken in the immediate vicinity of the element at *z*_1_ = 0.1λ for all cases except circular deepening (*z*_1_ = 0.4λ).

It should be noted that as the height of the element increased, the main focus was formed inside the element, but at the same time, the formation of the second focus outside the element was noticed. An increase in the height of the relief unevenly affected the length of the light segments and their shape, as well as the overall diffraction pattern. This was especially noticeable at r = 2λ for all types of micro-defects. 

It should be noted that with an increase in height, focusing mainly took place on the cylinders. The depression was not used for focusing at h > λ, and the square protrusion did not allow a focal spot to be obtained on the optical axis with an acceptable intensity value.

The best results were obtained for silicon cylinders. In particular, for a cylindrical protrusion with r = λ at h = 0.4λ, a compact focal spot with FWHM = 0.28λ (0.58 of the maximum intensity) was formed in the immediate vicinity of the element. The best result for a cylinder with r = 2.0λ was obtained for h = 1.5λ (0.58 of the maximum intensity). However, we chose the case h = λ for further research. In this case, for a silicon cylinder with r = 1.0λ (FWHM = 0.29λ), the focal intensity was 0.8 of the maximum, and for a cylinder with r = 2.0λ it was 0.51 (FWHM = 0.41λ). The values were obtained at *z*_1_ = 0.1λ. 

**Figure 6:**
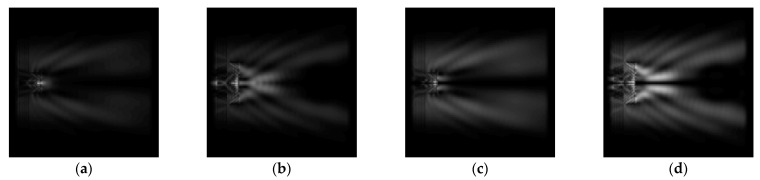
Comparison of silicon cylinders with circular polarization, plane xz, h = λ: (**a**) r = λ, “−” circular polarization, (**b**) r = 2λ, “−” circular polarization, (**c**) r = λ, “+” circular polarization, (**d**) r = 2λ, “+” circular polarization.

The action of silicon micro-cylinders for circular polarization is compared in Figure 6. The polarization recognition for a micro-cylinder with a radius of r = 2.0λ was demonstrated at a greater distance from the element, so we used it for further comparison.

The results of polarization recognition for a silicon cylinder with r = 2λ and a silicon diffraction axicon with NA = 0.25 at the same element height h = λ are shown in Table 5. Cross sections for the cylinder were taken at a distance *z*_1_ = 0.5λ, for an axicon at a distance *z*_1_ = 0.2λ (recognition beyond 0.4λ is difficult for an axicon). 

As can be seen from Table 5, we could visually distinguish all the considered types of polarizations, although it was difficult to recognize the azimuthal polarization for the axicon (the intensity of the central peak was 0.08).

We compared the results obtained for h = 0.2 and h = λ. Increasing the height resulted in better focusing for the silicon cylinder. In particular, the result of the minimum cross section for FWHM for x-linear polarization improved by 8.8% (FWHM(|) = 0.31λ and FWHM(|) = 0.34λ, respectively), the result for y-linear polarization improved by 23.8% (FWHM(–) = 0.32λ and FWHM(–) = 0.42λ, respectively), for “−” circular polarization they improved by 17.7% (FWHM = 0.37λ and FWHM = 0.45λ, respectively), and for azimuthal polarization they improved by 21.3% (FWHM = 0.37λ and FWHM = 0.47λ, respectively). 

The sizes of the focal spots for “−” circular polarization and azimuthal polarization for silicon cylinder were the same, but for the latter the intensity value in the center were two times lower than for “−” circular polarization.

An improvement in focusing was also observed for the diffractive axicon. In particular, the focal spot for the “−” circular polarization became smaller by 21.3% than with h = 0.2λ (FWHM = 0.48λ and FWHM = 0.61λ, respectively).

**Figure 7:**
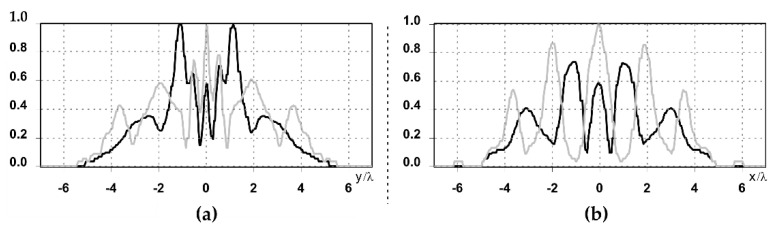
The cross sections for the cases in Table 5, circular protrusion with r = 2.0λ (black line) and diffractive axicon with NA = 0.25 (gray line), y-linear polarization (**a**) along the narrow part of the focal spot and (**b**) along the wide part of the focal spot.

The graphs with cross-sections (circular protrusion and diffractive axicon with NA = 0.25) for diffraction patterns from Table 5 for y-linear polarization are shown in Figure 7.

**Figure 8:**
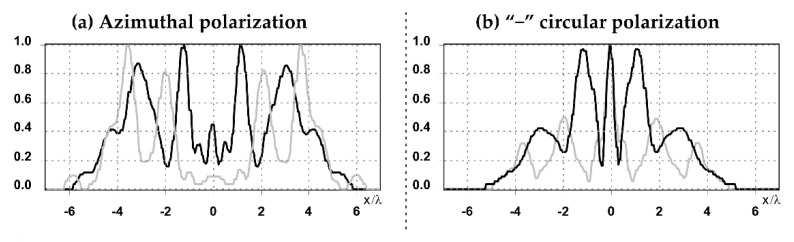
The cross sections for the cases in Table 5, circular protrusion with r = 2.0λ (black line) and diffractive axicon with NA = 0.25 (gray line): (**a**) azimuthal polarization, (**b**) “−” circular polarization.

The more pronounced side lobes for the circular protrusion were clearly visible in comparison with the diffractive axicon case. The graphs with cross-sections (circular protrusion and diffractive axicon with NA = 0.25) for diffraction patterns from Table 5 for azimuthal and “−” circular polarizations are shown in Figure 8.

It is clearly seen in Figure 8 that a more pronounced focal spot with a higher intensity was observed when focusing with circular protrusion with r = 2.0λ, than for focusing with a diffractive axicon with NA = 0.25.

**Figure 9:**
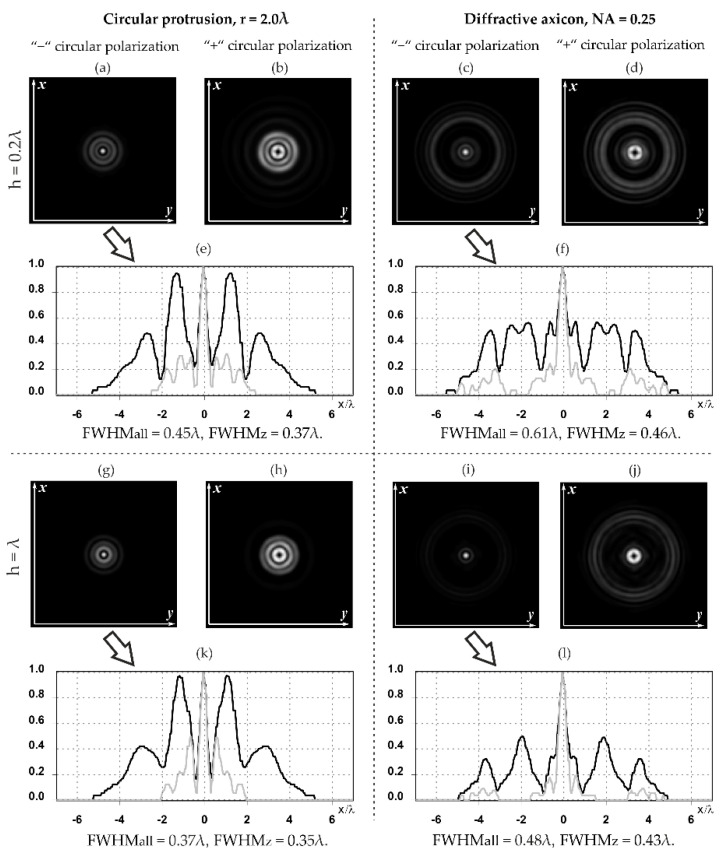
The redistribution of the longitudinal component of the electric field at circular polarization for a circular protrusion, r = 2.0λ, *z*_1_ = 0.5λ—(**a**,**b**,**g**,**h**) and a diffractive axicon, NA = 0.25, *z*_1_ = 0,2λ—(**c**,**d**,**i**,**j**); the graphs show a comparison of the total intensity (black) and the intensity of the longitudinal component of the electric field (gray)—(**e**,**f**,**k**,**l**).

It is known that the longitudinal component of the electric field is enhanced when a phase singularity is introduced into homogeneous–polarized radiation [12,26,30]. We demonstrated the redistribution of the energy of the longitudinal component from the periphery to the optical axis (Figure 9).

The distance from the relief (Figure 9) was chosen, similarly to Table 5. The maximum relative contribution of the longitudinal component of the electric field on the optical axis was obtained for circular polarization when a first-order vortex phase singularity of the opposite sign was introduced into the beam (“−” circular polarization). It is seen in Figure 8 that the size of the focal spot formed by the longitudinal component of the electric field decreased with an increase in the height of the dismounted elements. The focal spot width for the total intensity of the electric field and for the intensity of the longitudinal component of the electric field with increasing heights of the considered optical elements became close.

## 4. Discussion

The difference in the formed intensity pattern is shown on the basis of comparative modeling of the diffraction of optical vortices of the first order (Laguerre–Gauss mode (0.1)) on a cylindrical protrusion and a deepening of the sub-wavelength radius. During diffraction on a cylindrical protrusion of laser radiation, focusing is observed near the surface of the element. One of the main features of diffraction at a depression is the formation of an interference pattern of radiation created by the edges of the depression. It is possible to unambiguously determine the type of surface micro-defect from the diffraction pattern: a square or circular protrusion and a circular depression. It is also possible to distinguish all considered types of polarization: x-linear, y-linear, “−” circular, “+” circular, radial, and azimuthal polarizations of laser radiation.

It was shown that the equations used to determine different types of polarization for a diffractive axicon (*n* = 1.46), with a numerical aperture close to the limiting one, work in a similar way for such a simple element of micro-optics as a silicon cylinder. The possibility of recognizing the considered types of polarization not only by a silicon diffraction axicon with NA = 0.95, but also by a silicon axicon with NA = 0.25 at a distance shorter than λ is also demonstrated. Moreover, a silicon cylinder with r = 2λ at a distance of 0.5λ from the element makes it possible to obtain a focal spot of comparable or smaller size than a diffraction axicon made of silicon with NA = 0.95.

As originally assumed, a change in the element height significantly affects the diffraction pattern. The research for micro-defects with “−” circular polarization showed that with increasing height, focusing mainly occurred on the cylinders, and the main maximum was formed inside, and waveguide effects were observed. However, the next maximum was formed outside the element. The depression should not be used for focusing at h > λ, and the square protrusion does not allow a focal spot to be obtained on the optical axis with an acceptable intensity value.

Further comparison of polarization recognition for a silicon cylinder with r = 2λ and a silicon diffraction axicon with NA = 0.25 at the same element heights h = λ and h = 0.2λ showed a better focusing of the cylinder as compared to the axicon. In particular, for a cylinder with a height h = λ, it was possible to reduce the focal spot size (FWHM) from 0.45λ (h = 0.2λ) to 0.37λ for “−” circular polarization, and from 0.47λ to 0.37λ for azimuthal polarization, respectively.

An improvement in focusing with increasing altitude is also shown for the diffractive axicon. In particular, the focal spot for the “−” circular polarization was reduced from 0.61λ (FWHM) at h = 0.2λ, to 0.48λ at h = λ.

The article results show that micro-cylinders (circular protrusion) height is also an important value for localization of intensity near the edge of the element and formation of photonic micro-jets.

The decreases in light spot size when the longitudinal electric field component was redistributed to the central part of the laser beam for diffractive axicons with a substrate refractive index *n* = 1.46 (NA = 0.95) were shown earlier numerically [11,12,26] and experimentally [30]. We showed an increase in the contribution of the longitudinal component to the total intensity of the central focal spot for both a silicon circular protrusion with r = 2.0λ and a silicon diffractive axicon with NA = 0.25 with increases in the height of the elements in this work.

Selecting the longitudinal component of the electric field is important in applications where high degrees of localization of focus areas are required, for example, in microscopy, material processing, micromanipulation and particle capture, the use of selectively sensitive materials, and others.

Theoretically, it can be assumed that the detection of biological micro-objects is also possible. Bacteria detection with thin wetting film lens-less imaging was demonstrated earlier where the detection of micro-objects as small as a few μm, e.g., *Bacillus subtilis* bacteria [67], was shown. The classification of biological micro-objects using optical coherence tomography is also well known; in [68] light back-scattered by a scattering substrate is used, and not by the cells directly.

In our case, biological micro-objects can be considered as protrusions. Their shapes are difficult to determine, but in principle, their detection is possible. Theoretically, it is possible to track object height when a biological micro-object lies on the surface. However, taking into account the research carried out, it is possible to estimate the object width; early research has shown that with an element diameter smaller than λ, the micro-defect (protrusion or depression) is practically not determined. Accordingly, with this type of input beam, it is possible to detect micro-objects larger than λ (larger than 1.55 μm), such as the Pithoviridae giant virus family, which is a viral particle in the form of a prolate spheroid with size up to 2.5 μm in length and 0.9 μm in diameter [69].

## 5. Conclusions

In this paper, we studied the diffraction of optical vortices on elementary micro-optics objects, such as micro-cylinders, in the form of a protrusion and depression, a square micro-protrusion. The focusing is compared with diffractive axicons with different numerical apertures. The propagation of light (3D) through the proposed optical elements was simulated using the finite difference time domain method.

The possibility of recognition by the considered optical elements of the polarization type (linear, circular, radial, azimuthal) of the input radiation is shown. Thus, micro-defects act as sensors for the polarization state of the illuminating beam. The result of focusing with a circular cylinder with a radius of r = 2λ was better than focusing by a diffractive axicon with NA = 0.25 (grating period 4λ) and comparable to focusing by a diffractive axicon with NA = 0.95. The radius of the central zone of the diffractive axicon with NA = 0.95 was substantially sub-wavelength (0.26λ), and it is more difficult to manufacture such an element than a conventional circular protrusion.

The possibility of sub-wavelength focusing is demonstrated when the height of the elements is changed. In particular, it is numerically shown that a silicon cylinder (protrusion) illuminated by a laser beam with a first-order vortex phase singularity with circular polarization forms a light spot with a minimum size FWHM = 0.28λ.

## Figures and Tables

**Table 1:** The diffraction of Laguerre–Gauss mode (0,1) by surface micro-defects in plane xz, |**E**|^2^, [−6.96λ; 6.96λ].

Polarization	r = 1.0λ	r = 2.0λ		
	Circular Protrusion	Circular Protrusion	Square Protrusion	Circular Deepening
x-linear	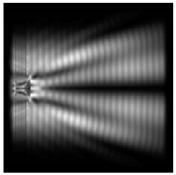	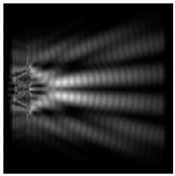	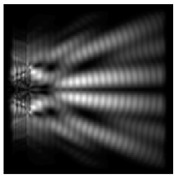	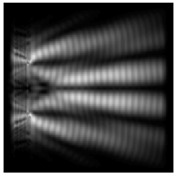
z-linear	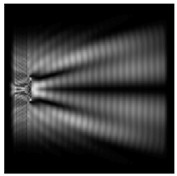	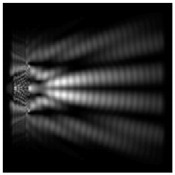	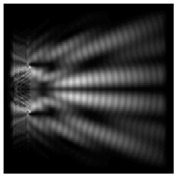	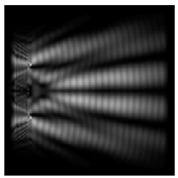
“−” circular	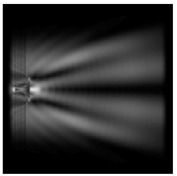	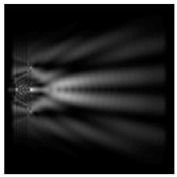	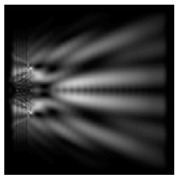	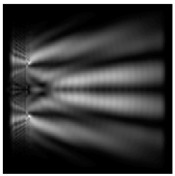
“+” circular	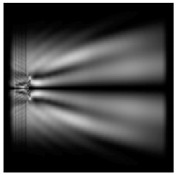	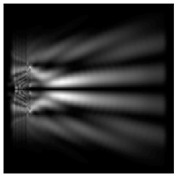	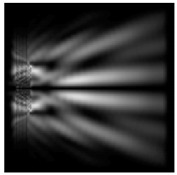	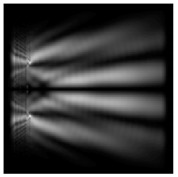
radial	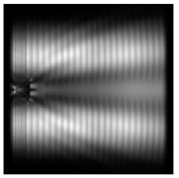	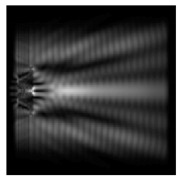	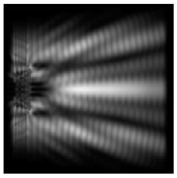	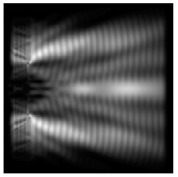
azimuthal	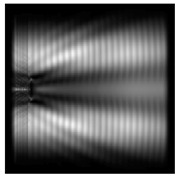	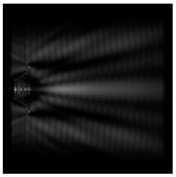	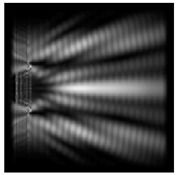	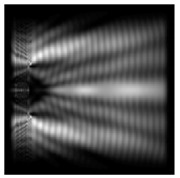

**Table 2:** The diffraction of Laguerre–Gauss mode (0,1) by surface micro-defects in plane xy (*z*_1_ = 0.5λ), |**E**|^2^, [−6.96λ; 6.96λ].

Polarization	r = 1.0λ	r = 2.0λ		
	Circular Protrusion	Circular Protrusion	Square Protrusion	Circular Deepening
x-linear	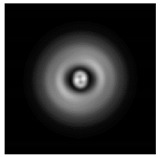 FWHM(|) = 1.62λ FWHM(–) = 1.25λ	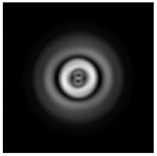 FWHM(|) = 0.34λ FWHM(–) = 0.91λ	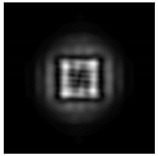	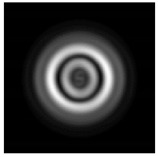 FWHM(|) = 3.7λ FWHM(–) = 3.3λ
y-linear	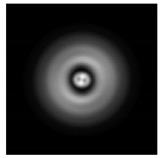 FWHM(|) = 1.28λ FWHM(–) = 1.5λ	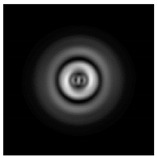 FWHM(|) = 0.78λ FWHM(–) = 0.42λ	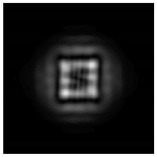	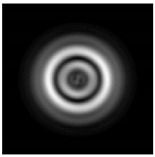 FWHM(|) = 3.4λ FWHM(–) = 2.4λ
“−” circular	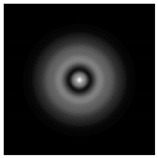 FWHM = 1.13λ	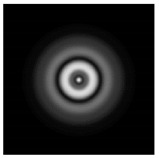 FWHM = 0.45λ	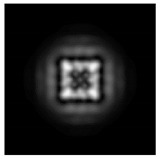	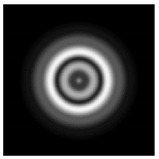 FWHM = 0.76λ
“+” circular	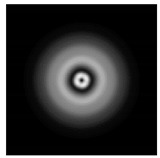	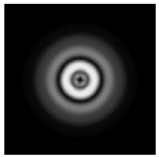	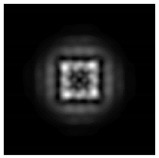	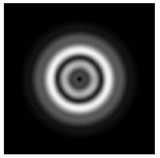
radial	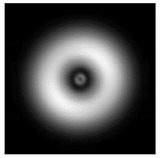	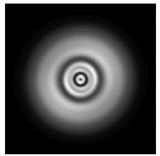	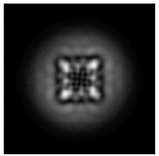	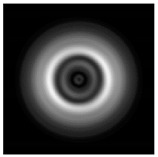
azimuthal	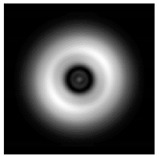 FWHM = 0.78λ	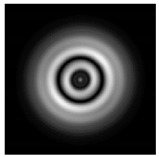 FWHM = 0.47λ	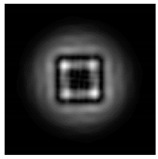	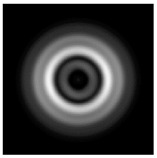 FWHM = 0.5λ

**Table 3:** The diffraction of Laguerre–Gauss mode (0,1) by diffractive axicons, |**E**|^2^, [−6.96λ; 6.96λ].

Polarization	NA = 0.25		NA = 0.95	
	Plane xz	Plane xy, *z*_1_ = 0.5λ	Plane xz	Plane xy, *z*_1_ = 0.5λ
x-linear	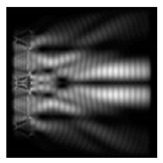	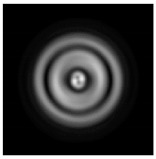 FWHM(|) = 1.65λ FWHM(–) = 1.28λ	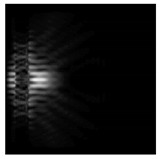	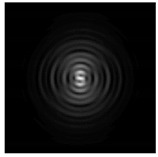 FWHM(|) = 0.43λ FWHM(–) = 0.85λ
y-linear	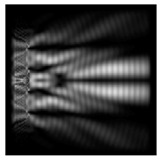	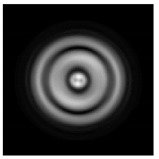 FWHM(|) = 1.34λ FWHM(–) = 1.54λ	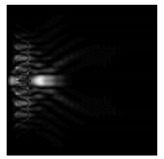	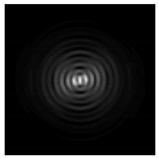 FWHM(|) = 0.78λ FWHM(–) = 0.45λ
“−”circular	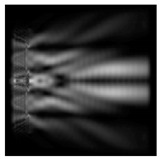	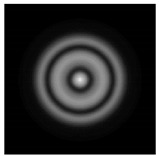 FWHM = 1.27λ	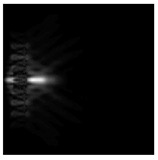	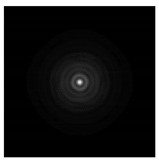 FWHM = 0.48λ
“+” circular	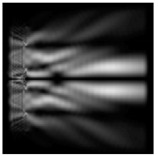	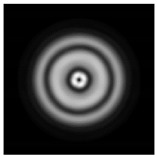	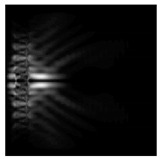	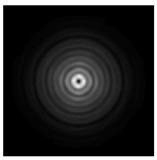
radial	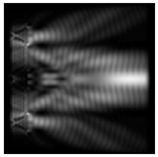	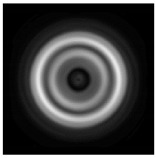	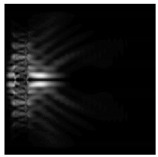	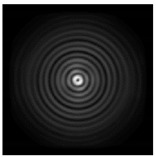
azimuthal	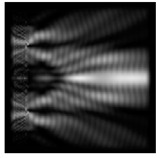	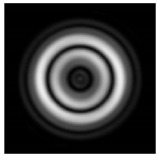 FWHM = 1.8λ	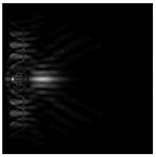	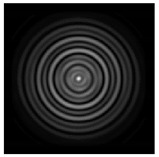 FWHM = 0.47λ

**Table 4:** The Laguerre–Gauss mode (0,1) diffraction by surface micro-defects with the variable height, plane xz, |**E**|^2^, [−6.96λ; 6.96λ].

Element Height	r = 1.0λ	r = 2.0λ		
	Circular Protrusion	Circular Protrusion	Square Protrusion	Circular Deepening
h = 0.2λ	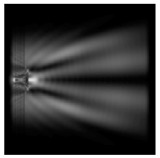 FWHM = 0.56λ	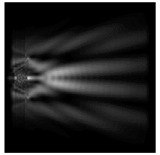 FWHM = 0.35λ	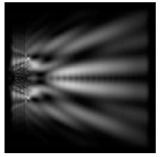	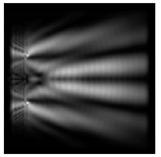 FWHM = 0.64λ
h = 0.4λ	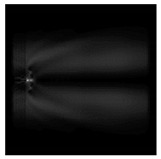 FWHM = 0.28λ	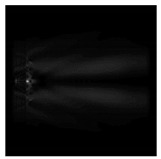 FWHM = 0.45λ	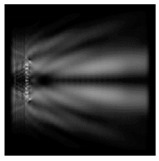	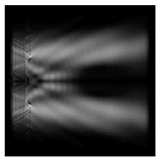
h = 0.5λ	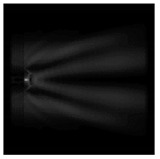 FWHM = 1.54λ	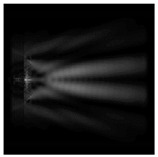 FWHM = 0.44λ	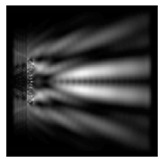	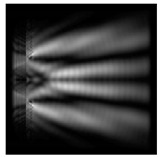 FWHM = 0.62λ
h = 1.0λ	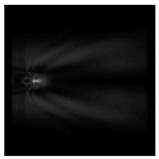 FWHM = 0.29λ	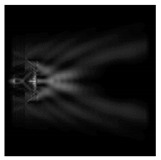 FWHM = 0.41λ	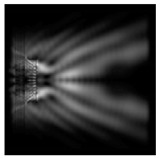	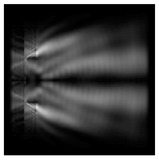
h = 1.5λ	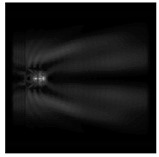 FWHM = 0.46λ	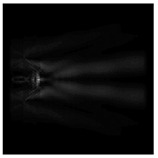 FWHM = 0.33λ	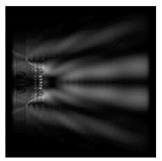	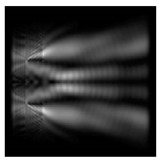
h = 2.0λ	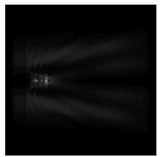 FWHM = 0.89λ	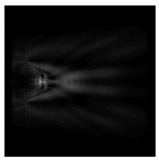 FWHM = 0.39λ	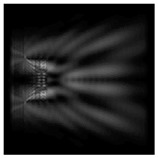	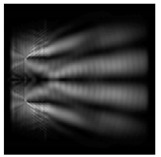

**Table 5:** The diffraction of Laguerre–Gauss mode (0,1) by a diffractive axicon (NA = 0.25) and silicon cylinder (protrusion, r = 2.0λ) at height h = λ, |**E**|^2^, [−6.96λ; 6.96λ].

Polarization	Circular Protrusion, r = 2.0λ		Diffractive Axicon, NA = 0.25	
	Plane xz	Plane xy, *z*_1_ = 0.5λ	Plane xz	Plane xy, *z*_1_ = 0.2λ
x-linear	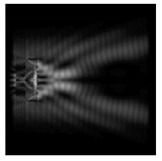	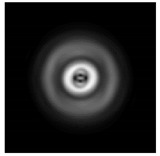 FWHM(|) = 0.31λ FWHM(–) = 0.74λ	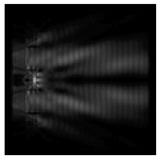	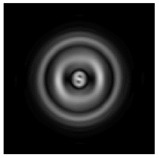 FWHM(|) = 0.45λ FWHM(–) = 1.09λ
y-linear	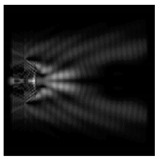	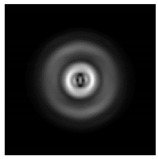 FWHM(|) = 0.71λ FWHM(–) = 0.32λ	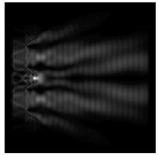	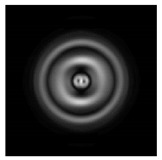 FWHM(|) = 0.98λ FWHM(–) = 0.43λ
“−” circular	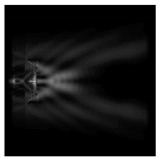	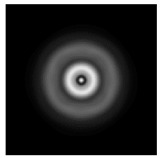 FWHM = 0.37λ	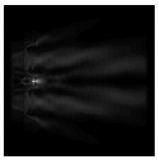	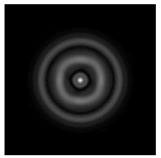 FWHM = 0.48λ
“+” circular	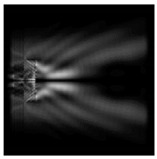	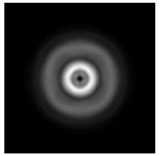	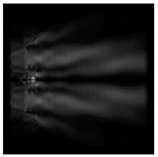	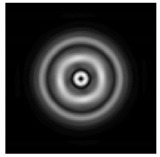
radial	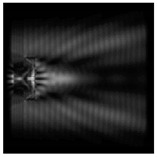	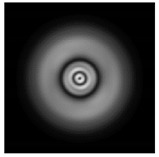	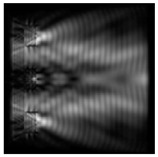	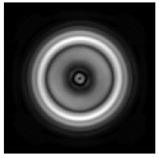
azimuthal	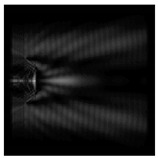	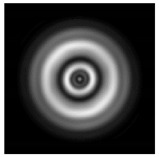 FWHM = 0.37λ	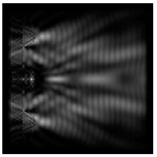	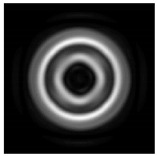 FWHM = 0.98λ

## Data Availability

The data presented in this study are available in this article.
